# Supplementary material for: Gene–Gene Interactions Reduce Aminoglycoside Susceptibility of Pseudomonas aeruginosa through Efflux Pump-Dependent and -Independent Mechanisms
Source: Antibiotics (Basel). 2023 Jan 11;12(1):152. doi: 10.3390/antibiotics12010152 (PMC9854422; doi:10.3390/antibiotics12010152)
Supplement: Supplementary file 1 [file antibiotics-12-00152-s001.zip › Supplementary Figures S1 and S2.pdf]

Figure S1

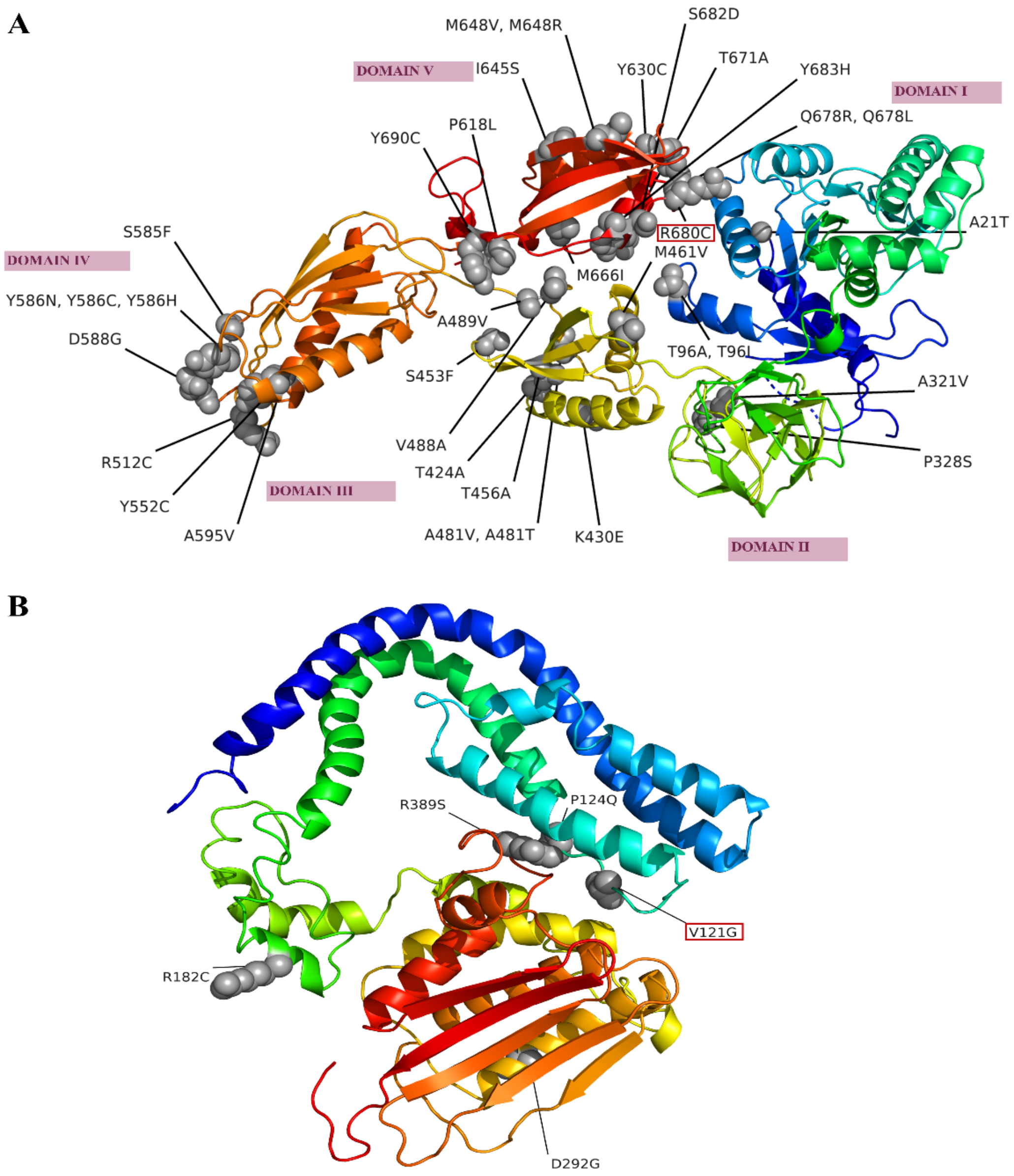

Figure S1. Mapping of frequent clinical variants in structural models of FusA1 and AmgS. Likely function-altering sequence variants present in two or more clinical isolates are shown with sidechains displayed in grey. **A.** Variants present in FusA1 (PDB Accession ID: 4FN5) and protein domains are represented. The location of the engineered residue (R680C) is shown. **B.** The partial structure of AmgS was predicted using Phyre2. The location of the engineered residue (V121G) is shown.

Figure S2

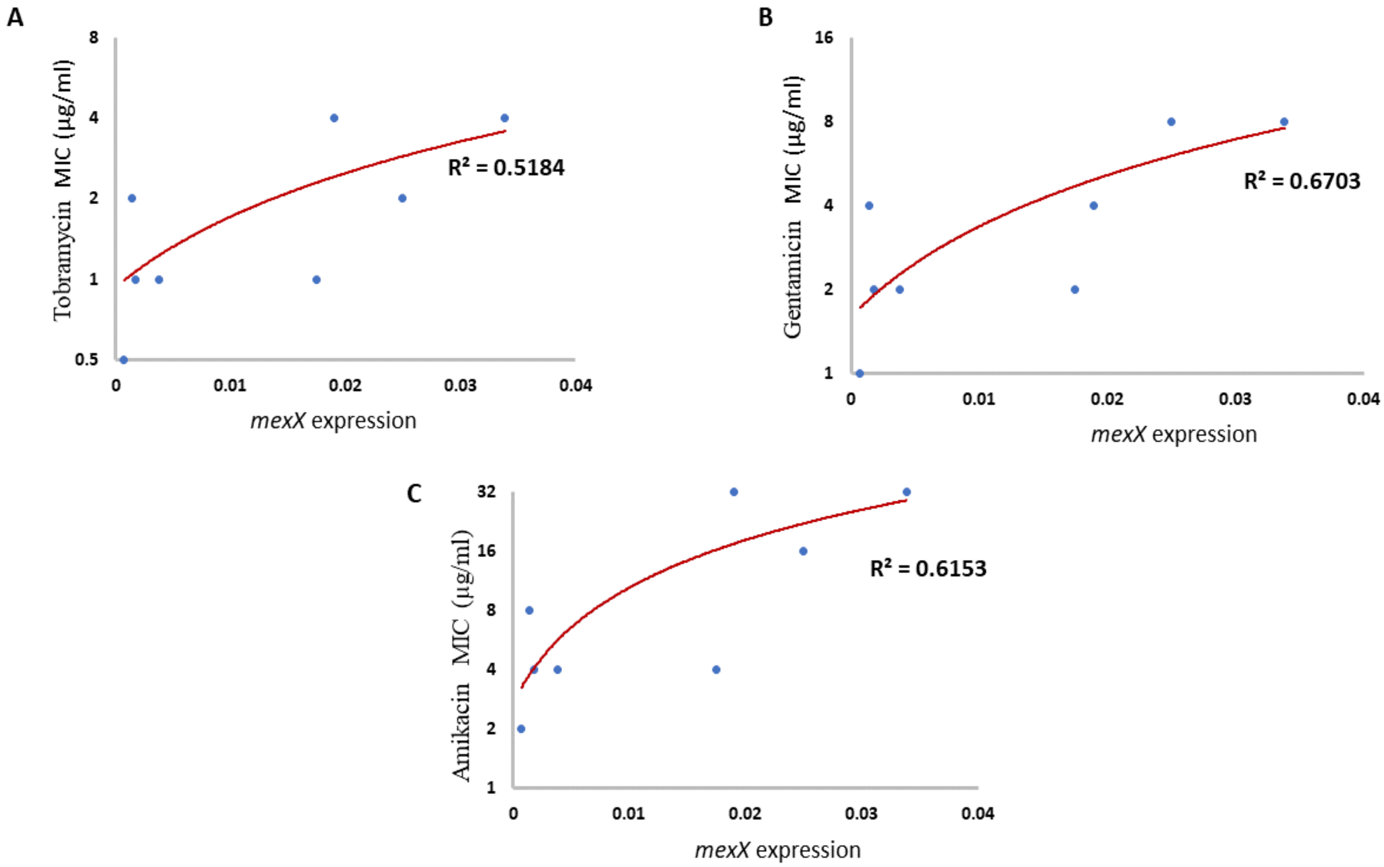

Figure S2. Correlation of aminoglycoside MICs with *mexX* expression. The MICs of engineered mutants were correlated with *mexX* expression and R^2 values are shown in the graphs. (A) tobramycin, (B) gentamicin and (C) amikacin.
