# Supplementary material for: Gene–Gene Interactions Reduce Aminoglycoside Susceptibility of Pseudomonas aeruginosa through Efflux Pump-Dependent and -Independent Mechanisms
Source: Antibiotics (Basel). 2023 Jan 11;12(1):152. doi: 10.3390/antibiotics12010152 (PMC9854422; doi:10.3390/antibiotics12010152)
Supplement: Supplementary file 1 [file antibiotics-12-00152-s001.zip › Supplementary Tables S1 and S2.pdf]

Table S1. Bacterial strains and plasmids used in the study

Bacterial Strains/ Plasmids	Genotype/ relevant characteristics	Reference
***Pseudomonas aeruginosa* reference strains**		
PAO1	Laboratory reference strain	[1]
**PAO1 mutants**		
Δ*mexZ*	Deletion of *mexZ* gene in PAO1	[2]
Δ*mexXY*	Deletion of *mexXY* genes in PAO1	[2]
mexY$_{G287S}$	Engineered mutation in the genome of PAO1 that replaced amino acid G with S at *mexY* codon 287	This study
fusA1$_{R680C}$	Engineered mutation in the genome of PAO1 that replaced amino acid R with C at codon 680 of *fusA1*	This study
fusA1$_{Y683D}$	PAO1 with spontaneous mutation that replaced amino acid Y with D at codon 683 of *fusA1*	[3]
fusA1$_{D327S}$	PAO1 with spontaneous mutation that replaced amino acid D with S at codon 327 of *fusA1*	[3]
amgS$_{V121G}$	Engineered mutation in the genome of PAO1 that replaced amino acid V with G at codon 121 of *amgS*	This study
Δ*mexZ mexY*$_{G287S}$	Δ*mexZ mexY*$_{G287S}$ double mutant	This study
Δ*mexZ fusA1*$_{R680C}$	Δ*mexZ fusA1*$_{R680C}$ double mutant	This study
Δ*mexXY fusA1*$_{R680C}$	Δ*mexXY fusA1*$_{R680C}$ double mutant	This study
Δ*mexZ fusA1*$_{Y683D}$	Δ*mexZ fusA1*$_{Y683D}$ double mutant	This study
Δ*mexZ fusA1*$_{D327S}$	Δ*mexZ fusA1*$_{D327S}$ double mutant	This study
Δ*mexZ amgS*$_{V121G}$	Δ*mexZ amgS*$_{V121G}$ double mutant	This study
Δ*mexXY amgS*$_{V121G}$	Δ*mexXY amgS*$_{V121G}$ double mutant	This study
mexY$_{G287S}$ *amgS*$_{V121G}$	*mexY*$_{G287S}$ *amgS*$_{V121G}$ double mutant	This study
mexY$_{G287S}$ *fusA1*$_{R680C}$	*mexY*$_{G287S}$ *fusA1*$_{R680C}$ double mutant	This study
fusA1$_{R680C}$ *amgS*$_{V121G}$	*fusA1*$_{R680C}$ *amgS*$_{V121G}$ double mutant	This study
Δ*mexZ amgS*$_{V121G}$ *fusA1*$_{R680C}$	Δ*mexZ amgS*$_{V121G}$ *fusA1*$_{R680C}$ triple mutant	This study
Δ*mexXY amgS*$_{V121G}$ *fusA1*$_{R680C}$	Δ*mexXY amgS*$_{V121G}$ *fusA1*$_{R680C}$ triple mutant	This study
Δ*mexZ fusA1*$_{R680C}$ *mexY*$_{G287S}$	Δ*mexZ fusA1*$_{R680C}$ *mexY*$_{G287S}$ triple mutant	This study
Δ*mexZ amgS*$_{V121G}$ *mexY*$_{G287S}$	Δ*mexZ amgS*$_{V121G}$ *mexY*$_{G287S}$ triple mutant	This study

fusA1$_{R680C}$ *amgS*$_{V121G}$ *mexY*$_{G287S}$	*fusA1*$_{R680C}$ *amgS*$_{V121G}$ *mexY*$_{G287S}$ triple mutant	This study
Δ*mexZ fusA1*$_{R680C}$ *amgS*$_{V121G}$ *mexY*$_{G287S}$	Δ*mexZ fusA1*$_{R680C}$ *amgS*$_{V121G}$ *mexY*$_{G287S}$ quadruple mutant	This study
**Strains of *Escherichia coli***		
JM83	*ara*, Δ(*lac-proAB*) *rpsL*(*strA*), φ80, *lacZ*ΔM15	[4]
ST18	S 17 λ*pir*Δ*hemA*	[5]
**Plasmids used in the study**		
pEX18Tc	pMB1 replicon, *oriT* ,*sacB*, Tc^R , allelic exchange vector	[6]
pEX18Tc::Δ*mexXY*	pEX18Tc derivative carrying *mexXY* (*PA2018-PA2019*) flanking regions	[2]
pEX18Tc::Δ*mexZ*	pEX18Tc derivative carrying *mexZ* (*PA2020*) flanking regions	[2]
pEX18Tc::*mexY*$_{G287S}$	pEX18Tc containing DNA fragment with a mutation that encodes amino acid S instead of G at residue 287 in MexY	This study
pEX18Tc:: *fusA1*$_{R680C}$	pEX18Tc containing DNA fragment with a mutation that encodes amino acid C instead of R at residue 680 in FusA1	This study
pEX18Tc:: *amgS*$_{V121G}$	pEX18Tc containing DNA fragment with a mutation that encodes amino acid G instead of V at residue 121 in AmgS	This study

Table S2. Primers used in the study.

Primers	Sequences	Reference
***mexXY* deletion Primers**		
*mexXY*_F1	5’ CCCCC GGTACC GAGTCGGCTGATG ACCTACA 3’	[2]
*mexXY*_R1	5'GGGGG TCTAGA GTACCGCTGTTCTTCCTGGT 3'	[2]
*mexXY*_F2	5’ CCCCC TCTAGATGTCCCTCGATTC GTGAACT 3’	[2]
*mexXY*_R2	5' GGGGG AAGCTT GCTCTACATCGAC GGCAAG 3'	[2]
***mexXY* deletion screening Primers**		
*PA2017*_F	5' GCA GCC TGT ACG TGG TCA 3'	[2]
*mexZ*_R	5' GGG TTT TCT GGG ATT CCT CT 3'	[2]
***mexZ* deletion Primers**		
*mexX*_F	5' CCCCC GAATTC GTTCTCGACGATCACCCACT 3'	[2]
*mexX*_R	5' GGGGG TCTAGA GGGTTTTCTGGGATTCCTCT 3'	[2]
*PA2022*_F	5' CCCCC TCTAGA CGCAGTTCTCCCTACCTGTT 3'	[2]
*PA2022*_R	5' GGGGG AAGCTT CGCAGTATCTGGCTGTCGTA 3'	[2]
***mexZ* deletion screening Primers**		
*mexZ_SR*_F	5' GTGTCCCTCGATTCGTGAAC 3'	[2]
*mexZ_SR*_R	5' CGTGAAGCTACCGTGACAGA 3'	[2]
mexY$_{G287S}$ **mutation engineering primers**		
*mexY*_F	5' GGGGAATTCCCTGTTCCGCAATCCGCATC 3'	This study
*mexY*_R	5'GGGGGATCCAGTCCTTCAGGGTGGCGAAG 3'	This study
mexY$_{G287S}$ **mutation screening primers**		
*mexY_scr*_F	5' CTCGTCCAACGTGTTGCAGG 3'	This study
*mexY_scr*_R	5' AACGCCGAGGTGTCATAGGG 3'	This study
fusA1$_{R680C}$ **mutation engineering primers**		
*fusA1*_F	5' GGGGG TCTAGA TTACTCGATGATCTTGGCAAC 3'	This study
fusA1_R	5' CCCCC AAGCTT GAAGCCGAGATCAAGGAAGG 3'	This study
fusA1$_{R680C}$ **mutation screening primers**		
*fusA1_scr*_F	5' GTATTCAACGTGCGAGGTGT 3'	This study

*fusA1_scr*_R	5' GTTCAAGATCGCTGCTTCCA 3'	This study
amgS$_{V121G}$ **mutation engineering primers**		
*amgS*_F	5'CCCCC GAATTC TTCTCCGTAAGCACGAGAGG 3'	This study
*amgS*_R	5' CCCCC TCTAGA AGCAGATGGATCGCCTTCTA 3'	This study
amgS$_{V121G}$ **mutation screening primers**		
*amgS_scr*_F	5' ATACACCTCGGCCATTTCAC 3'	This study
*amgS_scr*_R	5' CTGATGAACGAGGACGTGAT 3'	This study
**RT-qPCR Primers**		
*mexX_RT*_F	5′ GGC CCT GGT CGC CCT ATT C 3′	[7]
*mexX_RT*_R	5′ TCC TCG TAC AGG CGA CGG 3′	[7]
clpX_F	5′ GTG GGC GAG GAT GTC GAG AAC 3′	[7]
clpX_R	5′ CGG TAC CCT CGA TGA GCT TCA G 3′	[7]
oprL_F	5′ CCA ACA GCG GTG CCG TTG A 3′	[7]
oprL_R	5′ GCC ATA TTG TAC TCG CGG GT 3′	[7]
**Universal primers**		
M13_F	GTA AAA CGA CGG CCA GT	Universal
M13_R	CAG GAA ACA GCT ATG AC	Universal

[a]Introduced restriction sites are underlined.

1. Holloway, B. Genetic recombination in *Pseudomonas aeruginosa*. *Journal of General Microbiology* **1955**, *13*, 572-581.

2. Thacharodi, A.; Lamont, I.L. Aminoglycoside resistance in Pseudomonas aeruginosa: the contribution of the MexXY-OprM efflux pump varies between isolates. *Journal of Medical Microbiology* **2022**, *71*, 001551.
3. Wardell, S.J.; Rehman, A.; Martin, L.W.; Winstanley, C.; Patrick, W.M.; Lamont, I.L. A large-scale whole-genome comparison shows that experimental evolution in response to antibiotics predicts changes in naturally evolved clinical *Pseudomonas aeruginosa*. *Antimicrob Agents Chemother.* **2019**, *63*, e01619-01619.
4. Yanisch-Perron, C.; Vieira, J.; Messing, J. Improved M13 phage cloning vectors and host strains: nucleotide sequences of the M13mpl8 and pUC19 vectors. *Gene* **1985**, *33*, 103-119.
5. Thoma, S.; Schobert, M. An improved Escherichia coli donor strain for diparental mating. *FEMS microbiology letters* **2009**, *294*, 127-132.
6. Hoang, T.T.; Karkhoff-Schweizer, R.R.; Kutchma, A.J.; Schweizer, H.P. A broad-host-range Flp-FRT recombination system for site-specific excision of chromosomally-located DNA sequences: application for isolation of unmarked Pseudomonas aeruginosa mutants. *Gene* **1998**, *212*, 77-86.
7. Martin, L.W.; Robson, C.L.; Watts, A.M.; Gray, A.R.; Wainwright, C.E.; Bell, S.C.; Ramsay, K.A.; Kidd, T.J.; Reid, D.W.; Brockway, B. Expression of Pseudomonas aeruginosa antibiotic resistance genes varies greatly during infections in cystic fibrosis patients. *Antimicrobial agents and chemotherapy* **2018**, *62*, e01789-01718.
